# Local availability of neonatal intensive care at rural hospitals with childbirth services

**DOI:** 10.1038/s41372-025-02518-4

**Published:** 2025-11-24

**Authors:** Emily C. Sheffield, Clara E. Busse, Julia D. Interrante, Sara C. Handley, Katy Backes Kozhimannil

**Affiliations:** 1 University of Minnesota Rural Health Research Center, University of Minnesota School of Public Health, Minneapolis, MN USA; 2 Division of Health Policy and Management, University of Minnesota School of Public Health, Minneapolis, MN USA; 3 Department of Pediatrics, University of Pennsylvania Perelman School of Medicine, Philadelphia, PA USA; 4 Leonard Davis Institute of Health Economics, University of Pennsylvania, Philadelphia, PA USA

**Keywords:** Public health, Health services

## Abstract

**Objective:**

To compare hospital- and county-level characteristics of rural US hospitals based on distance to the nearest neonatal intensive care unit (NICU).

**Study design:**

Cross-sectional analysis using data from a survey conducted March-August 2021 with administrators and maternity unit managers at rural hospitals with childbirth services (N = 89).

**Results:**

Few hospitals had a locally available NICU (onsite: *n* = 5, 5.6%; <10 miles: *n* = 0; 10–29 miles away: *n* = 5, 5.6%). Most were located ≥30 miles away from the nearest NICU (30–60 miles: *n* = 29, 32.6%; >60 miles: *n* = 50, 56.2%). All Critical Access Hospitals and hospitals in noncore (less populated) counties were ≥30 miles from NICUs. Hospitals further from NICUs more often had smaller birth volumes, higher proportions of Medicaid-paid births, fewer beds, and higher county-level proportions of lower income or unemployed residents.

**Conclusion:**

Most surveyed rural hospitals did not have locally available NICUs, and characteristics of those that did suggest more hospital and community resources.

## Introduction

Proximity to and receipt of risk-appropriate neonatal care are important determinants of health outcomes for infants [1–5]. For infants with higher acuity clinical needs or complex diagnoses, such as those with unexpected illness after birth, those born preterm, or those with congenital anomalies, such care is available in neonatal intensive care units (NICUs) [6, 7]. It is well established that hospitals with NICUs have been shown to have lower mortality rates for infants born preterm (< 37 weeks’ gestation) and those with very low birth weights (< 1500 g) compared to hospitals with childbirth services but without NICUs [4, 8, 9].

Hospitals in rural communities have limited capacity to offer NICU care, and rural communities have less access to these services [10]. Prior work shows that rural residents with multiple gestation pregnancies or who give birth preterm are less likely to give birth at a hospital with a NICU if one is not available within 30 miles [11]. Amidst rising infant mortality rates [12, 13] and declining access to childbirth care [14, 15] in the US—both of which are elevated for rural residents [12, 14, 16]—it is important to understand the proximity of NICU care for residents of rural communities.

The objective of this analysis was to characterize the local availability of and proximity to neonatal intensive care at surveyed rural hospitals with childbirth services in the US. We examine the reported distances from surveyed hospitals to the nearest NICU (onsite, <10 miles away, 10–29 miles away, 30–60 miles away, or >60 miles away) and compare rural hospitals in these distance categories across hospital- and county-level characteristics.

## Methods

### Data

Data for this cross-sectional analysis came from a web-based survey we conducted with hospital administrators and maternity unit managers at rural hospitals in the US between March and August 2021, which addressed many aspects of rural hospital-based childbirth services. Rural hospitals were defined as those in counties designated nonmetropolitan core-based statistical areas by 2010 Office of Management and Budget definitions [17]. Using a validated, previously published method combining multiple data sources (the American Hospital Association Annual Survey, the Centers for Medicare & Medicaid Services Provider of Services file, and primary source reviews of hospital websites and news stories), childbirth service availability (inclusive of basic obstetric and well infant care) was identified at all short-term acute care hospitals in the US [18]. The survey frame of hospitals in rural counties included (1) all hospitals that closed their childbirth units between 2010 and 2018; (2) a 20% sample of hospitals with childbirth services in 2018 located in rural counties with a majority non-Hispanic White population; and (3) all hospitals with childbirth services in 2018 in rural counties without a majority non-Hispanic White population, to ensure adequate representation of rural counties with larger proportions of Black, Indigenous, and/or Hispanic residents. Additional details about the survey design can be found in previous publications [19–21]. Some hospitals that were included in the survey frame because they had childbirth services in 2018 had closed their childbirth services by the time they were surveyed in 2021. Of the 292 surveyed hospitals that had childbirth services at the time of the survey, 93 responded (32% response rate).

### Measures

Distance to neonatal intensive care was asked among hospitals with childbirth services at the time of the survey with the following question: “How far away from your hospital is the closest Neonatal Intensive Care Unit (NICU)?” Respondents could select from the following options: We have an on-site NICU; <10 miles; 10–29 miles; 30–60 miles; >60 miles; or I don’t know. Hospitals with missing responses to this item were excluded from the analysis (*n* = 4).

Additional hospital-level data from the survey incorporated in the analysis included: distance to the next-nearest hospital with obstetric services, percent of births that were paid by Medicaid, and birth volume (in 2019) (Appendix Table 1). We also examined hospital-level characteristics from the 2021 American Hospital Association Annual Survey including Critical Access Hospital status, hospital system membership, hospital control (government, nonfederal; government, federal; nongovernment, not-for-profit; for-profit), and the number of staffed hospital beds.

We examined county-level characteristics from the 2023 Area Health Resources Files including rural county type (micropolitan counties (those with an urbanized town of 10,000–50,000 residents) or noncore counties (those without an urbanized town of at least 10,000 residents) based on 2020 Core-Based Statistical Area), urban adjacency (2013 Urban Influence Codes), 5-year estimates of the proportion of the county population living below the Federal Poverty Level (2017-2021), the proportion of births that were low birth weight ( < 2500 g; 2021), and 3-year estimates of the proportion of births that were preterm ( < 37 weeks’ gestation; 2019–2021). We also examined proportions of county populations that were unemployed from the 2018 Local Area Unemployment Statistics program and median household income in 2021 from the 2023 County Health Rankings file.

All continuous variables were dichotomized as greater than or equal to versus less than the median value in the final study sample.

### Analysis

We calculated the numbers and proportions of respondent hospitals in each of the NICU distance categories to describe the distribution of rural hospitals based on their reported distance to the nearest NICU. We then calculated the numbers and proportions of rural hospitals across hospital- and county-level characteristics, stratified by the reported NICU distance categories. We compared hospitals in the NICU distance categories across these characteristics using Fisher’s exact tests. All analyses were conducted in Stata v. 18.0 (College Station, TX).

This study was designated exempt from review by the University of Minnesota Institutional Review Board; informed consent did not apply. All methods were performed in accordance with the relevant guidelines and regulations.

## Results

**Fig. 1:**
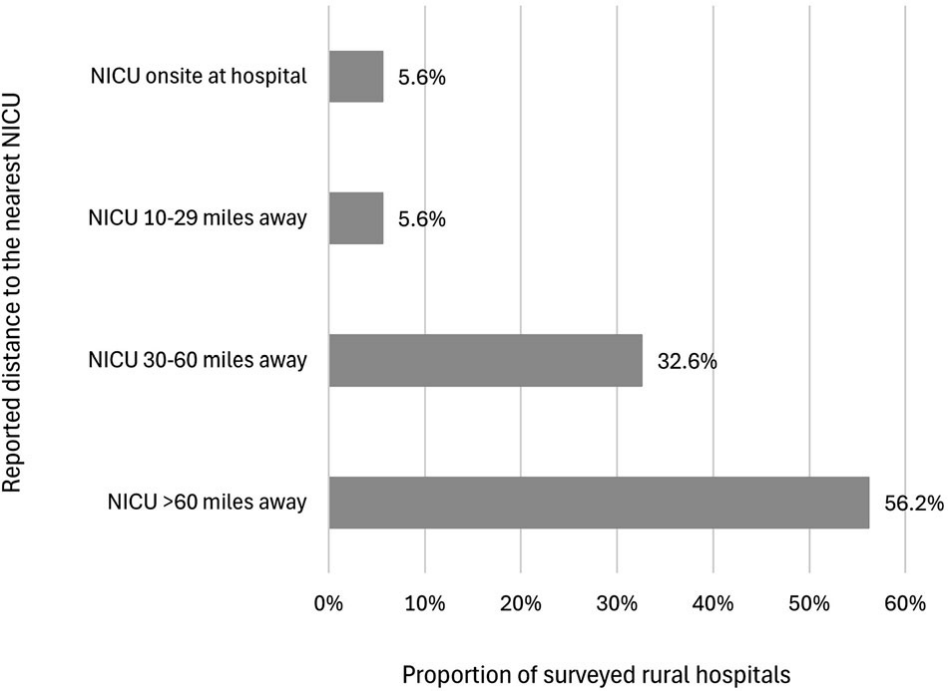
Distance to the nearest neonatal intensive care unit (NICU) among rural hospitals with childbirth services in 2021 (*n* = 89). No respondents reported an offsite NICU < 10 miles away from their hospital.

There were 89 rural hospitals with childbirth services in 2021 included in the analysis. The majority of respondents (56.2%; *n *= 50/89) identified that the nearest NICU was more than 60 miles away from their hospital, and an additional 33% of respondents (*n* = 29/89) reported that the nearest NICU was 30–60 miles away (Fig. 1). Few respondents identified a NICU being onsite at their rural hospital (5.6%; *n* = 5/89) or, separately, that the nearest NICU was 10–29 miles away from their hospital (5.6%; *n* = 5/89).

Hospital-level characteristics across the NICU distance categories are shown in Table 1. We found that all Critical Access Hospitals in this study were located ≥30 miles from the nearest NICU: 39% (*n* = 12/31) were 30–60 miles away from the nearest NICU and 61% (n = 19/31) were more than 60 miles away. Among hospitals with fewer beds (<49 facility beds), 36% (*n* = 16/44) were between 30 and 60 miles away and 59% (*n* = 26/44) were at least 60 miles away from the nearest NICU. Nearly all (*n* = 21/23; 91%) respondents who reported being >60 miles from the next-nearest hospital with obstetric services were also >60 miles away from the nearest NICU, with just 9% (*n* = 2/23) reporting that they had an onsite NICU at their hospital. The majority of hospitals with ≥62% Medicaid-paid births (median cut-point) did not have locally available neonatal intensive care: 36% (*n* = 15/42) were located 30–60 miles away and 60% (*n* = 25/42) were >60 miles away from the nearest NICU. We also found that very few hospitals with a lower than median birth volume (<274 births) had a NICU less than 30 miles away (*n* = 2/44; 4.6%), whereas all hospitals with an onsite NICU (*n* = 5/5) had a birth volume of 274 births or more.

**Table 1:** Hospital-level characteristics of rural hospitals with childbirth services in 2021, based on distance to the nearest neonatal intensive care unit (*n* = 89).

	Distance to nearest neonatal intensive care unit n (%^a^)				
	NICU onsite at hospital	NICU 10–29 miles away	NICU 30–60 miles away	NICU >60 miles away	*p* value^b^
	N (%)^a^ *N* = 5	N (%)^a^ *N* = 5	N (%)^a^ *N* = 29	N (%)^a^ *N* = 50	
***Hospital-level characteristics***					
Critical Access Hospital status					
Yes	0 (0.0)	0 (0.0)	12 (38.7)	19 (61.3)	0.114
No	5 (8.6)	5 (8.6)	17 (29.3)	31 (53.5)	
Hospital system membership					
Yes	3 (6.5)	3 (6.5)	16 (34.8)	24 (52.2)	0.838
No	2 (4.7)	2 (4.7)	13 (30.2)	26 (60.5)	
Hospital control					
Government, nonfederal	0 (0.0)	1 (4.8)	10 (47.6)	10 (47.6)	0.695
Government, federal	0 (0.0)	0 (0.0)	0 (0.0)	3 (100.0)	
Nongovernment, not-for-profit	4 (7.7)	3 (5.8)	16 (30.8)	29 (55.8)	
For-profit	1 (7.7)	1 (7.7)	3 (23.1)	8 (61.5)	
Hospital bed number^c^					
<49 beds	1 (2.3)	1 (2.3)	16 (36.4)	26 (59.1)	0.287
≥49 beds	4 (8.9)	4 (8.9)	13 (28.9)	24 (53.3)	
Distance to next-nearest hospital with obstetric services					
<10 miles away	0 (0.0)	0 (0.0)	0 (0.0)	3 (100.0)	<0.001
10–29 miles away	3 (11.5)	4 (15.4)	8 (30.8)	11 (42.3)	
30–60 miles away	0 (0.0)	1 (2.7)	21 (56.8)	15 (40.5)	
>60 miles away	2 (8.7)	0 (0.0)	0 (0.0)	21 (91.3)	
Percent Medicaid-paid births^c,d^					
<62% Medicaid-paid births	5 (12.2)	3 (7.3)	11 (26.8)	22 (53.7)	0.278
≥62% Medicaid-paid births	0 (0.0)	2 (4.8)	15 (35.7)	25 (59.5)	
Birth volume^c^					
<274 births	0 (0.0)	2 (4.6)	18 (40.9)	24 (54.6)	0.069
≥274 births	5 (11.1)	3 (6.7)	11 (24.4)	26 (57.8)	

No respondents reported an offsite NICU < 10 miles away from their hospital. All data for hospital-level characteristics were from 2021 except for birth volume, which was reported for 2019 because of the COVID-19 pandemic.

*NICU* neonatal intensive care unit.

^a^Row percents.

^b^Fisher’s exact tests.

^c^Value represents the median of the final study sample for each characteristic.

^d^*n* = 6 hospitals were missing data for percent Medicaid-paid births and are not included in the table for this variable (*n* = 83 for percent Medicaid-paid births).

With respect to county-level sociodemographic characteristics by NICU distance categories (Table 2), we found that all hospitals in this analysis located in more rural, noncore counties (*n* = 32/32)—including those both adjacent (*n* = 17) and not adjacent (*n* = 15) to a metropolitan area—were at least 30 miles away from the nearest NICU. All hospitals in this analysis with an onsite NICU (*n* = 5/5) as well as all hospitals with a NICU 10–29 miles away (*n* = 5/5) were located in micropolitan counties. Among those with an onsite NICU, 80% (*n* = 4/5) were in micropolitan counties that were not urban-adjacent. The majority of hospitals in counties with greater than the median proportion of the population living below the Federal Poverty Level (≥14%) were >60 miles away from the nearest NICU (*n* = 30/45; 66.7%). Similarly, among hospitals located in counties with lower than the median household income (<$46,703), most reported that their nearest NICU was between 30 and 60 miles away (*n* = 16/44; 36.4%) or >60 miles away (*n* = 26/44; 59.1%).

**Table 2:** County-level sociodemographic and neonatal characteristics of rural hospitals with childbirth services in 2021, based on distance to the nearest neonatal intensive care unit (*n* = 89).

	Distance to nearest neonatal intensive care unit n (%^a^)				
	NICU onsite at hospital	NICU 10–29 miles away	NICU 30–60 miles away	NICU > 60 miles away	*p* value^b^
	N (%)^a^ *N* = 5	N (%)^a^ *N* = 5	N(%)^a^ *N* = 29	N(%)^a^ *N* = 50	
***County-level sociodemographic characteristics***					
Rural county type and urban adjacency					
Micropolitan, urban-adjacent	1 (3.7)	3 (11.1)	11 (40.7)	12 (44.4)	0.006
Micropolitan, not urban-adjacent	4 (13.3)	2 (6.7)	4 (13.3)	20 (66.7)	
Noncore, urban-adjacent	0 (0.0)	0 (0.0)	11 (64.7)	6 (35.3)	
Noncore, not urban-adjacent	0 (0.0)	0 (0.0)	3 (20.0)	12 (80.0)	
Proportion of population below the Federal Poverty Level^c^					
<14%	5 (11.4)	3 (6.8)	16 (36.4)	20 (45.5)	0.051
≥14%	0 (0.0)	2 (4.4)	13 (28.9)	30 (66.7)	
Proportion of population unemployed^c^					
<4.5%	4 (9.3)	3 (7.0)	16 (37.2)	20 (46.5)	0.252
≥4.5%	1 (2.2)	2 (4.4)	13 (28.3)	30 (65.2)	
Median household income^c^					
<$46,703	0 (0.0)	2 (4.6)	16 (36.4)	26 (59.1)	0.142
≥$46,703	5 (11.1)	3 (6.7)	13 (28.9)	24 (53.3)	
***County-level neonatal characteristics***					
Proportion low birth weight ( < 2500 grams)^c,d^					
<7.6%	3 (6.7)	3 (6.7)	15 (33.3)	24 (53.3)	1.000
≥7.6%	2 (4.7)	2 (4.7)	14 (32.6)	25 (58.1)	
Proportion preterm births ( < 37 weeks’ gestation)^c,d^					
<11.8%	5 (11.4)	3 (6.8)	13 (29.6)	23 (52.3)	0.245
≥11.8%	0 (0.0)	2 (4.7)	16 (37.2)	25 (58.1)	

No respondents reported an offsite NICU < 10 miles away from their hospital. Data for county-level characteristics were measured in the following years: rural county type (2020); urban adjacency (2013); proportion of population below the Federal Poverty Level (2017-2021); proportion of population unemployed (2018); proportion preterm births (2019–2021); and median household income and proportion low birth weight (2021).

*NICU* neonatal intensive care unit.

^a^Row percents.

^b^Fisher’s exact tests.

^c^Value represents the median of the final study sample for each characteristic.

^d^*n* = 2 hospitals were missing data for proportion low birth weight and proportion preterm births and are not included in the table for these variables (*n* = 87 for proportion low birth weight and proportion preterm births).

Regarding county-level neonatal characteristics, surveyed hospitals in each NICU distance category were similar with respect to the proportion of low birth weight births (≥7.6% median cut-point) (Table 2). Among hospitals in counties with ≥11.8% (median cut-point) preterm births, most reported being 30–60 miles (*n* = 16/43; 37.2%) or >60 miles away (*n *= 25/43; 58.1%) from the nearest NICU. Notably, among rural hospitals that reported having an onsite NICU, all (*n* = 5/5) were located in counties where the population median was at or above the Federal Poverty Level, at least the median household income, and below the median proportion of preterm births, and 80% (*n* = 4/5) were in counties with lower than the median proportion of unemployed residents.

## Discussion

Access to NICU care is critically important for high-risk infants, and timing of access to care can affect quality and outcomes [22, 23]. Rural infants face greater risks of mortality than urban infants [12], and our analysis shows that having a locally available NICU was rare in rural communities with hospital-based childbirth services. Among surveyed rural hospitals, we found that most did not have locally available NICU care (onsite or <30 miles away), and more than half reported that their nearest NICU was >60 miles away. As increased distance to NICU care is associated with worse perinatal outcomes [2], infants born at rural facilities without locally available NICU care may face greater risks of morbidity and mortality.

Though most comparisons across NICU distance categories were not statistically significant at conventional levels (*p* < 0.05), likely due to small sample sizes, there were some meaningful trends across hospital- and county-level characteristics. In particular, hospitals with onsite NICUs or a NICU < 30 miles away tended to be located in facilities or counties that may be better resourced. Specifically, hospitals with a locally available NICU tended to have more beds, lower proportions of Medicaid-paid births, and higher birth volumes, and none were designated as Critical Access Hospitals. In addition, these hospitals were more often located in communities with characteristics associated with better socioeconomic conditions. Such hospitals were more often located in micropolitan (vs. noncore) rural counties and in counties with lower proportions of the population living below the Federal Poverty Level, lower proportions of unemployed residents, and higher median household incomes, compared with hospitals that were located further away from the nearest NICU (≥30 miles away).

Our findings are consistent with prior literature indicating a potential relationship between individual- and community-level socioeconomic resources and proximity to a NICU. One study found that women with Medicaid insurance or no insurance at childbirth, women living in rural noncore counties, and women living in socioeconomically disadvantaged counties (those with lower than the median annual household income or those with <30% of the county population having attained at least a college degree) were more likely to be located farther away from their nearest hospital with neonatal intermediate or intensive care [24]. In a study of rural residents with multiple gestation pregnancies or preterm births, women who did not have access to a local NICU (within 30 miles) were found to be significantly less likely to give birth at a hospital with a NICU if they were insured by Medicaid or uninsured at childbirth, compared to privately insured women [11]. Similarly, a study of high-risk births in California found that having Medicaid or any other public insurance (vs. private insurance) was associated with a lower likelihood of giving birth in a hospital with a NICU [25].

Many rural hospitals and communities do not have sufficient resources or patient volume to establish or safely sustain a NICU. Rural hospitals with low birth volumes are likely to face similar challenges in sustaining NICU services as they do in maintaining basic childbirth services (e.g., high costs of specialized equipment and personnel, workforce recruitment, and clinical skill maintenance [19]). Neonatal specialists predominantly practice in urban areas [26], where there are sufficient patient volumes and hospital resources to ensure maintenance of clinical skills and availability of necessary interventions. Prior literature has consistently documented lower risks of adverse outcomes for high-risk infants when they are cared for in hospitals that have more experience (e.g., greater patient volume) and appropriate resources (intermediate or intensive care). This has been demonstrated among very low birth weight (VLBW, <1500 g) infants cared for in higher-level hospitals with higher-VLBW infant volumes [8] and among moderate and late preterm infants (32–36 weeks’ gestation) cared for in lower-level units with higher moderate and late preterm infant volumes [5]. Given these challenges and realities, efforts to add or maintain NICU services at hospitals with smaller birth volumes, including those in rural areas, necessitate attention to both care quality and infant outcomes.

Given capacity limitations in rural hospitals, thoughtful and targeted efforts are needed to improve availability of and access to NICU care for rural infants. Some countries, such as Portugal, have addressed this issue via robust perinatal regionalization efforts and subsequently reported a substantial decrease in neonatal and infant mortality [27]. Other strategies to improve rural access to NICU care could include expanding high-risk patient referral systems and transfer policies. This includes both maternal (*in utero*) transport for infants who are identified as high-risk prenatally and appropriate for maternal transport [1] as well as infants whose need for transfer to higher-acuity services arises during or after birth [28, 29]. Additional opportunities include efforts to improve the availability of more advanced neonatal care by increasing local clinician capacity. For example, investment in simulation training for staff at rural hospitals without NICUs can help build and reinforce local clinicians’ skills and comfort with resuscitating and stabilizing infants while awaiting transfer to neonatal intensive care [30, 31]. Similarly, emergency medical services personnel, including those in rural communities, who may transport obstetric patients and neonates to hospitals with NICUs, should have specialized skills in caring for these patients [28, 32]. Expanding the use of clinician-to-clinician telemedicine consultations with neonatal specialists may also help support rural clinicians in hospitals without NICUs who are transiently managing ill infants [33–35]. Such strategies may specifically support hospitals and communities with fewer resources and mitigate the potential risks associated with limited local access to NICU care for rural infants.

Though not all infants need access to neonatal intensive care, access to and receipt of risk-appropriate neonatal care is important for high-risk infants who may need an advanced level of care [1], including infants born with congenital anomalies [36, 37], those born preterm [4], or those born with low birth weight [8]. While some infants are identified as high-risk prenatally, for others, the need for advanced neonatal care arises at the time of birth (e.g., infants with brain injuries at childbirth, such as hypoxic ischemic encephalopathy, who may need therapeutic hypothermia [38]), underscoring the importance of having nearby advanced care. In addition, as the availability of hospital-based obstetric care in rural communities continues to decline [14, 15], access to prenatal care services may also decline [39–41], which may contribute to delays or gaps in identifying high-risk rural infants while they are in utero. As prior studies suggest that longer distances to perinatal care may increase the risk of an infant having a NICU admission or poor outcome [2, 3], and rural residents tend to have less access to perinatal care than their urban counterparts [21, 42–44], families in rural and remote communities face distinctive risks around the time of childbirth, including increased risks of infant mortality [12, 45]. While not all rural hospitals have the resources or need to establish or safely sustain a NICU, examining the hospital- and county-level characteristics associated with local availability of neonatal intensive care in rural communities can help inform efforts to improve access to needed care for rural infants and to reduce the risk of adverse health outcomes for high-risk infants in these communities [1, 24, 46].

### Limitations

This study contributes new information to the literature on the availability of neonatal intensive care in rural US communities. The main variable of interest in this analysis, distance to the nearest NICU, was reported by survey respondents, who may have over- or underestimated the distance from their hospital facility to the nearest NICU. Additionally, the survey did not include information on driving time (accounting for geography, roads, or weather), nor hospital systems (the nearest hospital with a NICU may not always be in the same hospital network or system). Small sample sizes within categories of NICU distance likely contributed to a lack of precision in our statistical analysis. While most comparisons of hospital- and county-level characteristics across NICU distance categories were not statistically significant at conventional levels (*p* < 0.05), many exhibited potentially meaningful trends, suggesting an uneven distribution of NICUs in rural communities. Finally, though our analytic sample includes rural hospitals in all four US Census regions [19], survey respondents were not representative of all rural hospitals in the US, so our findings may not be generalizable to all rural hospitals or communities.

## Conclusion

Having access to risk-appropriate neonatal care is important for the health of infants in rural communities. In this descriptive study, we found that most surveyed rural hospitals lacked locally available NICU care, and that hospitals with local access to these higher acuity services (those with onsite NICUs or a NICU <30 miles away) are hospitals with more resources than those located further away from the nearest NICU. Infants born in rural communities with fewer resources and higher perinatal health risks may also be further away from NICU care, and efforts to improve birth outcomes for rural infants should account for these access challenges.

## Supplementary information

Appendix Table 1. Survey questions among hospitals with childbirth services at the time of the survey

## Data Availability

The data used to create the sampling frame for this survey include information that is subject to a data use agreement and cannot be made available to others. The survey instrument has been published as an online supplemental document in prior publications [19]. Analytic code can be provided by the corresponding author, on request.
