# Supplementary material for: Local availability of neonatal intensive care at rural hospitals with childbirth services
Source: J Perinatol. 2025 Nov 24;46(5):853–9. doi: 10.1038/s41372-025-02518-4 (PMC13190270; doi:10.1038/s41372-025-02518-4)
Supplement: Supplementary file 1 — Appendix Table 1. Survey questions among hospitals with childbirth services at the time of the survey [file 41372_2025_2518_MOESM1_ESM.docx]

**Appendix Table 1.** Survey questions among hospitals with childbirth services at the time of the survey

| ***Distance to neonatal intensive care*** |
| --- |
| How far away from your hospital is the closest Neonatal Intensive Care Unit (NICU)?   1. We have an on-site NICU 2. < 10 miles 3. 10-29 miles 4. 30- 60 miles 5. > 60 miles 6. I don't know |
| ***Distance to next-nearest hospital with obstetric services*** |
| How far from your hospital is the next-nearest hospital that provides inpatient labor and birth services?   1. < 10 miles 2. 10-29 miles 3. 30- 60 miles 4. > 60 miles |
| ***Percent Medicaid-paid births*** |
| Approximately what percentage of the births at your hospital are paid for by… (Medicaid; Private or commercial insurance; Self-pay or uninsured; Indian Health Service; Tricare; Other) |
| ***Birth volume*** |
| Approximately how many births occurred in your hospital in **2019**? |
